# Information About the Optimism of a Placebo/Nocebo Provider and Placebo/Nocebo Side Effects

**DOI:** 10.3389/fpsyg.2020.608595

**Published:** 2021-01-14

**Authors:** Carina Schlintl, Anne Schienle

**Affiliations:** Department of Clinical Psychology, University of Graz, Graz, Austria

**Keywords:** placebo/nocebo side effects, personality, optimism, provider, recipient

## Abstract

**Background:**

Research has demonstrated that personality characteristics, such as optimism are associated with placebo/nocebo responding. The present study investigated whether written information about the optimism of a placebo/nocebo provider can influence the occurrence of reported placebo/nocebo side effects.

**Method:**

We analyzed data from 201 females (mean age = 26 years) who participated in a “clinical study on a new massage oil with stone clover extract.” The oil (sunflower oil) was introduced as either eliciting a negative side effect (unpleasant itching; “nocebo oil”) or a positive side effect (pleasant tingling; “placebo oil”). The administration of the oil was combined with written information about the maker of the product. The oil maker was either portrayed as a very optimistic person or no personal information was provided (only the company name). The participants had no personal contact with the experimenter and received all materials and instructions per post.

**Results:**

The participants reported more frequent and intense itching when they received a nocebo suggestion compared to a placebo suggestion. Positive tingling sensations were reported more frequently than itching but did not differ between the placebo/nocebo conditions. Information about the optimism of the oil maker was associated with a lower frequency of reported side effects (adverse and beneficial).

**Conclusion:**

This study demonstrated that it is sufficient to provide participants with written information about an inert substance to elicit the suggested side effect. Information about the provider’s optimistic personality did not specifically influence reported side effects. Future studies should focus on how to adapt written information about a drug/product to minimize adverse side effects and to maximize positive side effects.

## Introduction

Placebos and nocebos are substances or interventions with no specific effect on the symptom being treated. While placebos improve a person’s condition (e.g., reduction of negative symptoms), nocebo treatment is associated with the occurrence of negative symptoms, the worsening of a condition, or the prevention of improvement (Moerman and Jonas, 2002; Häuser et al., 2012). This definition has been specified by Faasse et al. (2019) who differentiate between primary placebo/nocebo effects and placebo/nocebo side effects. For example, when using a primary nocebo the potential adverse outcome is framed as the focal effect of the inert treatment, whereas a nocebo side effect refers to an adverse outcome that is ancillary to the typically beneficial outcome of the inert treatment.

The effects of placebos and nocebos have been conceptualized as “context effects” (Miller and Kaptchuk, 2008; Wager and Atlas, 2015). Important aspects of the context around placebo/nocebo treatment are social factors. The treatment is usually carried out as part of social interactions between healthcare providers and patients/clients. These interactions are shaped by the characteristics of both the providers and the recipients (e.g., for a review see Jakšić et al., 2013).

For example, personality factors such as optimism of the placebo recipients can influence their reactions to the inert treatment (e.g., Geers et al., 2005, 2010; Morton et al., 2009; Zhou et al., 2019; Kern et al., 2020). Trait optimism is an individual difference variable that reflects the extent to which individuals hold generalized favorable expectancies for their future. Higher levels of optimism are correlated prospectively with better subjective well-being in times of adversity or difficulty (i.e., controlling for previous well-being; Carver et al., 2010). A review by Kern et al. (2020) indicated that optimistic people show increased placebo responsivity. In a placebo study by Zhou et al. (2019), the reduction of pain unpleasantness was modulated by the interaction between expectancy and dispositional optimism. The latter finding illustrates that placebo/nocebo responding depends on both personality and situational factors. In line with this idea, studies found that pessimists were more likely than optimists to follow a nocebo expectation, whereas optimists showed greater benefit from the placebo condition (e.g., Geers et al., 2005; Hyland et al., 2007).

Additionally, optimism and confidence of the placebo provider can influence the placebo response (e.g., Kaptchuk et al., 2008; Howe et al., 2017; Daniali and Flaten, 2019; Gaab et al., 2019). Shapiro (1969) introduced the term “iatroplacebogenics” to describe placebo effects produced by health care professionals in the context of medical and psychotherapeutic treatment. These effects include the attitude to the patient and the attitude to the treatment (Feldman, 1956; Uhlenhuth et al., 1966; Shapiro, 1969; Gracely et al., 1985). Similarly, Brody (1997) has suggested that physicians can be “walking placebos” to stimulate positive changes in their patients through their attitudes and personality. In line with this idea, an early study by Uhlenhuth et al. (1966) demonstrated that patients who received an anxiolytic drug showed greater improvement when their doctors expressed a positive, enthusiastic attitude toward the medication compared to an uncertain, experimental attitude. In a more recent study, Kaptchuk et al. (2008) examined the effects of placebo acupuncture on irritable bowel syndrome. They found that the moderate effects of the placebo could almost be doubled when provided by a friendly and empathetic practitioner. Placebos that were administered during psychological treatment (“a video with green dots that activates positive emotional schemata”) improved the mood of the participants, but only when provided by a trustworthy and optimistic experimenter (Gaab et al., 2019).

In the mentioned studies, the placebos were provided in a supportive atmosphere. The placebo providers attempted to create a positive relationship with the placebo recipients and expressed their optimistic attitude concerning treatment success. These types of social interactions are time-consuming and often cannot be realized in the healthcare system.

Therefore, the present study aimed to investigate whether it is sufficient to provide written information about the optimistic personality of the placebo provider to influence the placebo/nocebo response. The placebo recipients had no personal contact with the experimenter. All materials and instructions were sent by post. The participants of the present study were invited to a “clinical study that tested a massage oil with stone clover extract.” The massage oil (sunflower oil) was either introduced as a substance with a negative side effect (unpleasant itching; “nocebo oil”) or a substance with a positive side effect (pleasant tingling sensation; “placebo oil”). The administration of the oil was combined with written information about who made the oil. The oil maker was either portrayed as a very optimistic person, or no personal information (only the company name) was mentioned. It was hypothesized that information about an optimistic oil maker would enhance positive skin sensations in the placebo condition, and reduce negative skin sensations in the nocebo condition.

## Materials and Methods

### Participants

A total of 245 females participated in this study. Inclusion criteria for the study were age over 18 years and female sex. We only tested females because of reported sex differences in placebo/nocebo responsivity (e.g., Vambheim and Flaten, 2017). Exclusion criteria included reported diagnoses of mental disorders and somatic diseases that might affect the responses to the oil (e.g., skin conditions). This led to the exclusion of five participants because of reported acne, neurodermatitis, and allergies. Furthermore, participants who did not complete the survey (*n* = 39) were excluded. Thus, data from *n* = 201 females (mean age = 26.16 years, SD = 7.83) were analyzed. The majority were university students (78%); the other participants were white-collar workers.

The study was approved by the Ethics Committee of the University (GZ. 39/75/63 ex 2019/20) and was performed following the Declaration of Helsinki. At the end of the study, all participants were fully debriefed.

### Procedure

The participants were invited to the study via announcements at the university and on social media. It was stated that a company that produced herbal products wanted to test their new massage oil. After obtaining written consent, the participants were asked to fill out two questionnaires via an online survey tool (LimeSurvey GmbH, Hamburg, Germany):

a) The short version of the Brief Symptom Inventory (BSI) (Spitzer et al., 2011) screens for mental problems. The BSI has 18 items (α = 0.87) and three subscales: Depression (six items; e.g., loss of interest, hopelessness; Cronbach’s α = 0.81), Anxiety (six items; e.g., nervousness, tension; α = 0.80), and Somatization (six items; e.g., dizziness, weakness; α = 0.73). The presence of symptoms is rated on five-point Likert scales ranging from 0 (not at all) to 4 (very strong). In the present sample, the t-scores for the BSI scores were all in the normal range (Depression: t-score = 0.57; Anxiety: t-score = 0.59; Somatization: t-score = 0.56; for mean scores see Supplementary Table 1).
b) The optimism/pessimism scale (Kemper et al., 2012) has two items: “Optimists are people who look to the future with confidence and usually expect good things. Please assess yourself: How optimistic are you in general?”; “Pessimists are people who are doubtful about the future and usually expect bad things. Please rate yourself: How pessimistic are you in general?” (Rating scale: 1 = not at all to 7 = very). In the total sample, the habitual optimism (*M* = 4.88, SD = 1.10) and pessimism (*M* = 2.90, SD = 1.18) did not differ from the mean scores reported by Kemper et al. (2012): optimism: *t*(1333) = −0.18, *p* = 0.857, pessimism: *t*(1333) = 0.95, *p* = 0.340). Ratings for optimism and pessimism were negatively correlated (*r* = −0.63, *p* < 0.001).

All participants who completed the online survey (which additionally asked for demographic information and diagnoses of somatic illness), received a package in the mail. This package contained a small glass bottle with sunflower oil and an information sheet. The bottle had a green label “*Melilotus officinalis*” and a dropper for application. The participants were instructed to apply 0.5 ml of the oil onto their left forearm (in an area with a diameter of 6 cm) and glide their digit finger softly over the area for 30 s. It was stated that the oil works quite quickly (“it takes 30 s to take effect”). The information sheet also included a suggestion about a specific side effect of the oil and information about the oil maker.

The suggested side effect was either pleasant (placebo side effect) or unpleasant (nocebo side effect). The suggestions were as follows:

a) Nocebo side effect: “This natural oil for your skin is extracted from the stone clover plant (*M. officinalis*). It has been developed for relaxation massages and promotes blood circulation. When applied, some users have noticed an unpleasant skin sensation: *itching*.”
b) Placebo side effect: “This natural oil for your skin is extracted from the stone clover plant (*M. officinalis*). It has been developed for relaxation massages and promotes blood circulation. When applied, some users have noticed a pleasant skin sensation: *tingling*.”

The placebo/nocebo suggestion was combined with one of two brief descriptions of the maker of the skin oil.

a) Optimistic maker: It was stated that the oil was made by Dr. Emilia Antonsini, a very dedicated physician and very optimistic person, who worked for a company producing natural medicine.
b) Company: In the control condition, only the company name was provided.

### Design

The study had an independent measures design with two variables: (1) Information about the oil MAKER (optimistic maker vs. company) and (2) SUGGESTION of side effect (placebo vs. nocebo). The participants were randomly allocated to one of four groups (PO: Placebo/optimistic maker, PC: Placebo/company, NO: Nocebo/optimistic maker, NC: Nocebo/company). The participants of the four groups did not differ in mean age, reported habitual optimism/pessimism, and BSI scores (all *p* > 0.28; see Supplementary Table 1).

**FIGURE 1:**
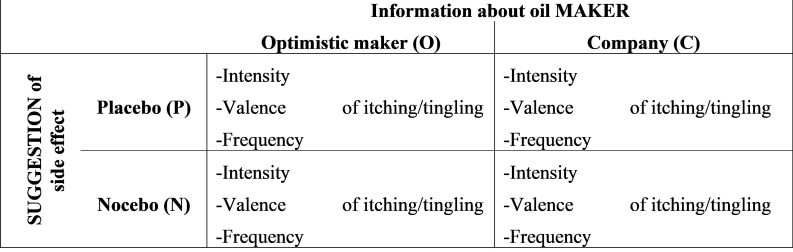
Study design.

Dependent variables were perceived valence, intensity, and frequency of the skin sensations itching and tingling (the suggested side effects). Valence and intensity were rated on nine-point scales (intensity: 1 = no sensation; 9 = very intense, valence: 1 = very unpleasant; 9 = very pleasant). For the study design see Figure 1.

The redness of the treated skin area was assessed as a control variable (as an indicator of the intensity of rubbing; 1 = no redness; 9 = intense redness). The intensity of observed redness was *M* = 1.13 (SD = 0.48) and did not differ between the experimental groups (*F*(3, 197) = 1.48, *p* = 0.221). All ratings were recorded via the online tool.

A pilot study (*n* = 50, *M* = 25.2 years, SD = 7.2) had indicated that the oil applied to the skin did neither elicit itching nor tingling sensations when the participants were correctly informed about the sunflower oil.

### Statistical Analysis

We computed 2 × 2 multivariate analyses of variance (MANOVAs) to test the effects of the between-subjects factors information about the oil MAKER (optimistic maker vs. company) and SUGGESTION (placebo vs. nocebo) on intensity and valence ratings for itching and tingling. Pillai’s trace (V) is reported as test statistic. Significant effects were followed up with ANOVAs. Effect sizes are expressed by part.η^2^ (partial eta squared).

To compare the frequency of reported itching/tingling (reported intensity > 1) between the four groups and different group combinations, we computed Chi^2^ tests. By combining the groups PO + PC and NO + NC, the effect of SUGGESTION (placebo vs. nocebo) can be tested; by combining the groups PO + NO and PC + NC, the effect of MAKER (optimistic maker vs. company) can be tested. Effect sizes are expressed by Cramer’s V.

To control for the possible influence of habitual optimism/pessimism of the participants on perceived intensity and valence of itching/tingling, we added these two factors separately as a covariate to a MANCOVA.

The conducted power analysis with G^∗^Power 3.1.9.2 (Faul et al., 2007) indicated that a minimum sample size of 102 would be needed to detect a medium effect size of *V* = 0.06 with a probability of 1–β = 0.80, α = 0.05 in the MANOVA for the between-subject factors SUGGESTION and MAKER on the dependent variables.

## Results

### Intensity of Itching/Tingling

The MANOVA revealed a significant main effect of SUGGESTION on the intensity of reported itching and tingling (*V* = 0.052, *F*(2, 196) = 5.40, *p* = 0.005, part.η2 = 0.052). The main effect of MAKER (*V* = 0.017, *F*(2, 196) = 1.69, *p* = 0.186, part.η2 = 0.017) and the interaction MAKER × SUGGESTION (*V* = 0.009, *F*(2, 196) = 0.86, *p* = 0.424, part.η2 = 0.009) were not significant. Follow-up ANOVAs showed that the Nocebo groups experienced more intense itching compared to the Placebo groups (*F*(1, 197) = 6.81, *p* = 0.010, part.η2 = 0.033). The reported intensity of tingling did not differ between the groups (*F*(1, 197) = 0.731, *p* = 0.394, part.η2 = 0.004).

### Valence of Itching/Tingling

The MANOVA revealed no significant main effects or interaction effects on the valence of itching and tingling (SUGGESTION: *V* = 0.017, *F*(2, 195) = 1.72, *p* = 0.181, part.η2 = 0.017; MAKER: *V* = 0.012, *F*(2, 195) = 1.19, *p* = 0.306, part.η2 = 0.012, SUGGESTION × MAKER: *V* = 0.008, *F*(2, 195) = 0.75, *p* = 0.475, part.η2 = 0.008; see Table 1).

**TABLE 1:** Means (M) and standard deviations (SD) of reported intensity and valence for the skin sensations (itching, tingling).

		Side Effect SUGGESTION	Information about MAKER	*M*	SD
Intensity	Itching	Placebo (P)	Optimistic (O)	1.07	0.33
			Company (C)	1.16	0.47
			Total Placebo (PO + PC)	1.12	0.40
		Nocebo (N)	Optimistic (O)	1.48	1.24
			Company (C)	1.31	0.67
			Total Nocebo (NO + NC)	1.39	0.98
		Total (P + N)	Optimistic (O)	1.26	0.89
			Company (C)	1.24	0.59
			Total (PO + PC + NO + NC)	1.25	0.75
	Tingling	Placebo (P)	Optimistic (O)	1.80	1.28
			Company (C)	2.33	1.84
			Total Placebo (PO + PC)	2.05	1.59
		Nocebo (N)	Optimistic (O)	1.80	1.73
			Company (C)	1.94	1.35
			Total Nocebo (NO + NC)	1.88	1.53
		Total (P + N)	Optimistic (O)	1.80	1.50
			Company (C)	2.13	1.61
			Total (PO + PC + NO + NC)	1.97	1.56
Valence	Itching	Placebo (P)	Optimistic (O)	5.28	1.66
			Company (C)	5.27	1.56
			Total Placebo (PO + PC)	5.27	1.61
		Nocebo (N)	Optimistic (O)	5.09	1.56
			Company (C)	5.00	1.30
			Total Nocebo (NO + NC)	5.04	1.42
		Total (P + N)	Optimistic (O)	5.19	1.61
			Company (C)	5.13	1.43
			Total (PO + PC + NO + NC)	5.16	1.52
	Tingling	Placebo (P)	Optimistic (O)	5.43	1.62
			Company (C)	5.84	1.57
			Total Placebo (PO + PC)	5.62	1.60
		Nocebo (N)	Optimistic (O)	5.24	1.49
			Company (C)	5.23	1.37
			Total Nocebo (NO + NC)	5.24	1.42
		Total (P + N)	Optimistic (O)	5.34	1.56
			Company (C)	5.52	1.49
			Total (PO + PC + NO + NC)	5.43	1.53

### Frequency of Itching/Tingling

#### Itching

**FIGURE 2:**
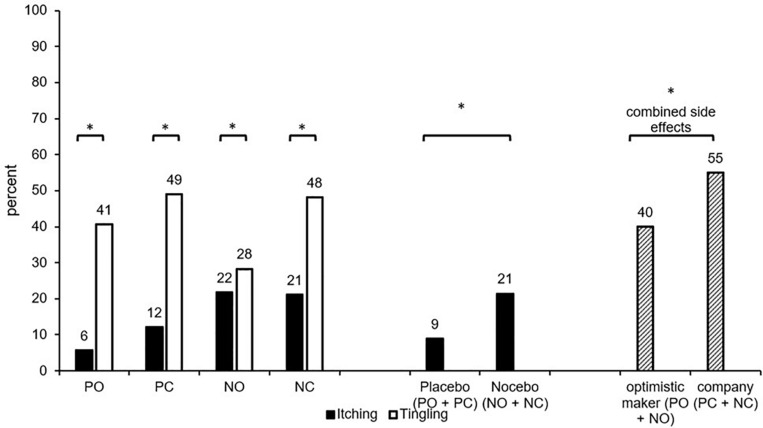
Percentage of participants who reported itching/tingling (intensity > 1) in the four conditions. PC, Placebo/company; PO, placebo/optimistic maker; NC, nocebo/company; NO, nocebo/optimistic maker; combined side effects (percentage of participants who reported itching and/or tingling). ^∗^significant difference (p < 0.05).

Itching was reported more often when the Nocebo side effect was suggested (NC + NO; *M* = 21%) compared to the Placebo side effect (PC + PO; *M* = 9%; Chi^2^(1) = 6.37; *p* = 0.012; *V* = 0.18). The information about the oil maker (optimistic maker: PO + NO: *M* = 13% vs. company: PC + NC: *M* = 17%; Chi^2^(1) = 0.58; *p* = 0.446; *V* = 0.05) did not influence itching. The comparison of the four groups (PC, PO, NC, NO) did not detect significant effects (Chi^2^(3) = 7.28; *p* = 0.063, *V* = 0.19; see Figure 2).

#### Tingling

The percentage of participants who reported tingling sensations did neither differ between the four groups (PC, PO, NC, NO; Chi^2^(3) = 5.37; *p* = 0.147; *V* = 0.16) nor any other group combination (all *p* > 0.05).

#### Combined Itching and Tingling

The percentage of participants who reported itching and/or tingling (combined placebo/nocebo side effects) did neither differ between the four groups (PC, PO, NC, NO; Chi^2^(3) = 4.54; *p* = 0.208; *V* = 0.15) nor between groups with Nocebo suggestions vs. Placebo suggestions of side effects (PC + PO: *M* = 49%; NC + NO: *M* = 46%; Chi^2^(1) = 0.14; *p* = 0.709; *V* = 0.03). However, INFORMATION about the oil maker influenced the combined side effects, which were less frequent when the maker was portrayed as optimistic (PO + NO: *M* = 40%) compared to the company information (PC + NC: *M* = 55%; Chi^2^(1) = 4.21; *p* = 0.040; *V* = 0.15, see Figure 2).

### Influence of Habitual Optimism/Pessimism of the Participants

The results of the MANCOVA are displayed in the Supplementary Table 2. Effects for optimism/pessimism were not statistically significant (all *p* > 0.324). The inclusion of the covariates did not change the results of the MANOVA (e.g., significant SUGGESTION effect on intensity of itching).

## Discussion

There is consensus that minimizing nocebo effects and maximizing placebo effects should lead to better treatment outcomes in clinical practice (Evers et al., 2018). Therefore, easy-to-implement interventions that achieve this goal are highly desirable. In the present investigation, we used a “minimal” nocebo/placebo approach with no personal contact between the provider and recipient of the inert treatment. The participants received written information about a skin oil that was introduced as either inducing “unpleasant itching” or “a pleasant tingling sensation” as a side effect. This information was combined with the written suggestion that a very optimistic person had made the oil or the participants received no personality-relevant information.

The chosen approach is similar to providing patients with written medication information (e.g., package inserts of prescription drugs). This type of information typically includes how the medication should be taken (dosage), desired effects, and possible side effects of the drug. It has been shown that written medication information provides a useful addition to counseling by healthcare professionals (Buck, 1998) and helps the patients to take the medication safely and appropriately.

The present study demonstrated that written information about the unpleasant side effect provided along with the oil was sufficient to elicit itching in one-fifth of the participants. When a nocebo suggestion was given, 21% of the participants reported itching. According to the European commission nomenclature for communicating the frequency of adverse effects of drugs (see Büchter et al., 2014), a probability of 1/10 is already considered a “very common” side effect. However, this adverse effect had a low average intensity. It has to be noted that placebo/nocebo studies on itching have revealed inconsistent results (for a review see Bartels et al., 2015). For example, Bartels et al. (2014) elicited itch electrically and found that neither conditioning nor verbal suggestion procedures applied individually induced significant placebo or nocebo effects. However, the combination of both methods was effective. In other studies of this research group, nocebo effects on itching were observed (Van Laarhoven et al., 2011) and could be minimized and even reversed by conditioning with verbal suggestions (Meeuwis et al., 2019).

In contrast to rather small nocebo effects on itching, the overall magnitude of the nocebo effect in studies on pain (reported increase in pain intensity) has been moderate to large (see meta-analysis by Petersen et al., 2014). These studies typically use noxious stimulation, whereas in the present study the stimulus (sunflower oil) was completely free of negative effects. Therefore, it is remarkable that weak itching symptoms occurred with substantial frequency. A somewhat similar effect has been reported by Colloca et al. (2008) who showed that non-painful tactile stimulation could be turned into a pain sensation via verbal nocebo suggestions in healthy participants. Moreover, Faasse et al. (2019) concluded in their overview article that nocebo side effects are weaker compared to the effects of primary nocebos.

The frequency of reported pleasant tingling sensations (42%) was considerably higher in the tested participants than itching (21%). This response can be expected because the sunflower oil had been introduced as a massage oil, which has a positive (placebo) connotation. Furthermore, in their review on the neuropsychophysiology of tingling, Tihanyi et al. (2018) have argued, that higher cognitive processes, such as attention and expectations play an important role in the generation of pleasant tingling sensations. For example, suggestion-induced tingling has been used in hypnotherapy to manage pain. Additionally, focused attention on a body part can give rise to spontaneous tingling (Tihanyi et al., 2018). It is perhaps of these focused attention effects that tingling was so frequently reported in the present investigation but did not differ between the nocebo and placebo conditions.

We were not able to demonstrate that information about the optimism of the placebo/nocebo maker specifically influenced the nocebo/placebo response of the participants. However, the general tendency to report side effects (both adverse and beneficial secondary effects) was lower in the conditions with personality-relevant information compared to the conditions where only the company name was mentioned. The participants were perhaps more reluctant to report side effects to a “real” person (“Emilia Antonsini”) than to an “abstract” company. Whether this effect is associated with the described personality of the provider cannot be decided based on the design of the present study. Additional conditions, such as the description of a pessimistic provider would be required.

As to the best of our knowledge, systematic personality assessments of successful placebo/nocebo providers have not been conducted so far. The majority of studies focused on state optimism of the providers, who created positive outcome expectations through their behavior (e.g., Kaptchuk et al., 2008; Gaab et al., 2019). Thus, these studies relied on the personal interaction of the placebo/nocebo provider with the recipient.

However, research on consumer behavior has demonstrated that “product beliefs” can influence product perception and liking. It is a common marketing strategy to associate a certain product/brand with a specific person (or personality). This information is usually transmitted through advertisements or labels and not via direct communication. For example, in a study by Robinson and Higgs (2012) participants reported reduced liking of orange juice when they believed that their in-group did not like the juice. Tinnermann et al. (2017) found that labeling an inert cream as an expensive medication led to stronger nocebo hyperalgesia than the label “inexpensive medication.” Thus, written information about the high price of the cream increased the risk of developing nocebo-related side effects. Crum et al. (2011) observed that identical milkshakes either labeled as high-calorie or low-calorie drinks received different ratings for perceived healthiness and elicited different hormonal (ghrelin) responses. However, this study did not find any significant label effects concerning subjective hunger after consumption or the tastiness of the milkshake. Thus, written product information (integrating social information) can be sufficient to change product evaluation (see Robinson and Higgs, 2012) but there are also boundary conditions (see Crum et al., 2011). Future studies on successful “placebo marketing” are therefore necessary.

In the present study, the reported dispositional optimism of the participants was not related to the intensity and valence of reported side effects. This is not in line with previous research (e.g., Geers et al., 2005, 2010; Hyland et al., 2007). In these studies, habitual pessimism was associated with increased nocebo responding, whereas optimists showed greater benefits from placebos. As mentioned before, optimism is a trait, which becomes particularly relevant in times of difficulties (Carver et al., 2010). The induced skin sensations in the present study however were evaluated as affectively neutral, on average.

As with any study, some limitations need to be acknowledged. We investigated healthy females. Therefore, the results cannot be generalized to other groups. The testing was conducted at home and not in a controlled lab environment. However, the participants were highly motivated to test the oil as reflected by a low dropout rate; 84% of the participants who received the oil by post completed the rating and often gave additional comments on the product (e.g., “is a little bit slimy,” “is not absorbed fast enough,” “where can I buy this product?,” “wonderful soft skin”). Generally, the intensity ratings for the skin sensations were low. In future studies, substances could be used that elicit the suggested effect of itching (see Bartels et al., 2015). Moreover, the description of the oil maker could be improved. The maker was generally characterized as a very optimistic person at the trait level but not concerning her attitude toward the side effect profile (the dependent variable of this study). Therefore, specification of the optimistic attitude (e.g., expressing confidence that the positive effects of the massage oil and not the negative side effects will dominate, or stressing that itching can be seen as a reminder of the massage oil having been absorbed) might be able to enhance the “optimism effect.”

## Conclusion

In conclusion, drug/product information on labels and package inserts are major sources of knowledge for patients/consumers. We demonstrated that written information about the side effect “itching” was sufficient to induce the suggested symptom in a substantial number of users. In contrast, a brief description of an optimistic nocebo/placebo provider and the optimism of the nocebo/placebo recipient did not specifically influence the placebo/nocebo response.

## Data Availability Statement

The raw data supporting the conclusions of this article will be made available by the authors, without undue reservation.

## Ethics Statement

The study was approved by the ethics committee of the University of Graz (GZ. 39/75/63 ex 2019/20) and was performed following the Declaration of Helsinki. All participants gave written informed consent. At the end of the study, all participants were fully debriefed.

## Author Contributions

AS designed the study. CS collected and helped to analyze the data. Both authors wrote the manuscript.

## Conflict of Interest

The authors declare that the research was conducted in the absence of any commercial or financial relationships that could be construed as a potential conflict of interest.

## Supplementary Material

Supplementary Table 1 Characteristics of the participants in the four groups. PO, placebo/optimistic maker; NO, nocebo optimistic maker; PC, placebo/company name; NC, nocebo/company name; BSI, Brief Symptom Inventory.

Supplementary Table 2 MANCOVA results. optimism/pessimism (of the participants); Suggestion: placebo vs. nocebo side effects; Maker: optimistic maker vs. company.
