# Supplementary material for: Information About the Optimism of a Placebo/Nocebo Provider and Placebo/Nocebo Side Effects
Source: Front Psychol. 2021 Jan 14;11:608595. doi: 10.3389/fpsyg.2020.608595 (PMC7841374; doi:10.3389/fpsyg.2020.608595)
Supplement: Supplementary Table 1 — Characteristics of the participants in the four groups. PO, placebo/optimistic maker; NO, nocebo optimistic maker; PC, placebo/company name; NC, nocebo/company name; BSI, Brief Symptom Inventory. [file Data_Sheet_1.PDF]

***Supplementary Table S1*: Characteristics of the participants in the four groups**

| | Condition | M | SD | F (df) | p |
|---|---|---|---|---|---|
| Mean age (years) | PC | 25.88 | 7.50 | 1.28 (3,197) | .284 |
| | PO | 27.91 | 10.48 | | |
| | NC | 25.31 | 6.94 | | |
| | NO | 25.39 | 4.81 | | |
| Habitual optimism | PC | 4.84 | 1.087 | .84 (3,197) | .475 |
| | PO | 5.07 | 1.163 | | |
| | NC | 4.81 | .886 | | |
| | NO | 4.76 | 1.251 | | |
| Habitual pessimism | PC | 3.70 | 1.373 | .98 (3,197) | .404 |
| | PO | 2.70 | 1.127 | | |
| | NC | 2.88 | 1.096 | | |
| | NO | 2.91 | 1.112 | | |
| BSI_Somatization | PC | 2.65 | 2.86 | .91 (3, 197) | .436 |
| | PO | 2.30 | 2.79 | | |
| | NC | 3.29 | 4.10 | | |
| | NO | 2.78 | 2.41 | | |
| BSI_Depression | PC | 2.69 | 2.79 | .54 (3, 197) | .657 |
| | PO | 2.91 | 3.72 | | |
| | NC | 3.25 | 3.83 | | |
| | NO | 2.43 | 2.56 | | |
| BSI_Anxiety | PC | 3.43 | 3.23 | .26 (3, 197) | .856 |
| | PO | 3.56 | 4.19 | | |
| | NC | 3.96 | 3.19 | | |
| | NO | 3.83 | 2.79 | | |

Footnote: (PO = placebo/ optimistic maker, NO = nocebo optimistic maker; PC = Placebo/ company name, NC = Nocebo/ company name); BSI: Brief Symptom Inventory)

***Supplementary Table S2*: MANCOVA results**

| | | Dependent variables: intensity_itching and intensity_tingling | | | |
|---|---|---|---|---|---|
| Effect | | V | F (df) | p | part.η2 |
| Optimism | Pillai-trace | ,012 | 1,135 (2, 195) | ,324 | ,012 |
| Suggestion | Pillai-trace | ,049 | 5,023 (2, 195) | ,007 | ,049 |
| Maker | Pillai-trace | ,018 | 1,765 (2, 195) | ,174 | ,018 |
| Suggestion * Maker | Pillai-trace | ,008 | ,773 (2, 195) | ,463 | ,008 |

| | | Dependent variables: valence_itching and valence_tingling | | | |
|---|---|---|---|---|---|
| Effect | | V | F (df) | p | part.η2 |
| Optimism | Pillai-trace | ,007 | ,712 (2, 194) | ,492 | ,007 |
| Suggestion | Pillai-trace | ,016 | 1,615 (2, 194) | ,201 | ,016 |
| Maker | Pillai-trace | ,012 | 1,169 (2, 194) | ,313 | ,012 |
| Suggestion * Maker | Pillai-trace | ,008 | ,751 (2, 194) | ,473 | ,008 |

| | | Dependent variables: intensity_itching and intensity_tingling | | | |
|---|---|---|---|---|---|
| Effect | | V | F (df) | Sig. | part.η2 |
| Pessimism | Pillai-trace | ,004 | ,402 (2, 195) | ,669 | ,004 |
| Suggestion | Pillai-trace | ,052 | 5,373 (2, 195) | ,005 | ,052 |
| Maker | Pillai-trace | ,018 | 1,798 (2, 195) | ,168 | ,018 |
| Suggestion * Maker | Pillai-trace | ,009 | ,916 (2, 195) | ,402 | ,009 |

| | | Dependent variables: valence_itching and valence_tingling | | | |
|---|---|---|---|---|---|
| Effect | | V | F (df) | Sig. | part.η2 |
| Pessimism | Pillai-trace | ,005 | ,456 (2, 194) | ,635 | ,005 |
| Suggestion | Pillai-trace | ,017 | 1,708 (2, 194) | ,184 | ,017 |
| Maker | Pillai-trace | ,013 | 1,277 (2, 194) | ,281 | ,013 |
| Suggestion * Maker | Pillai-trace | ,009 | ,859 (2, 194) | ,425 | ,009 |

Footnote: optimism/pessimism (of the participants); Suggestion: placebo vs. nocebo side effects; Maker: optimistic maker vs. company
